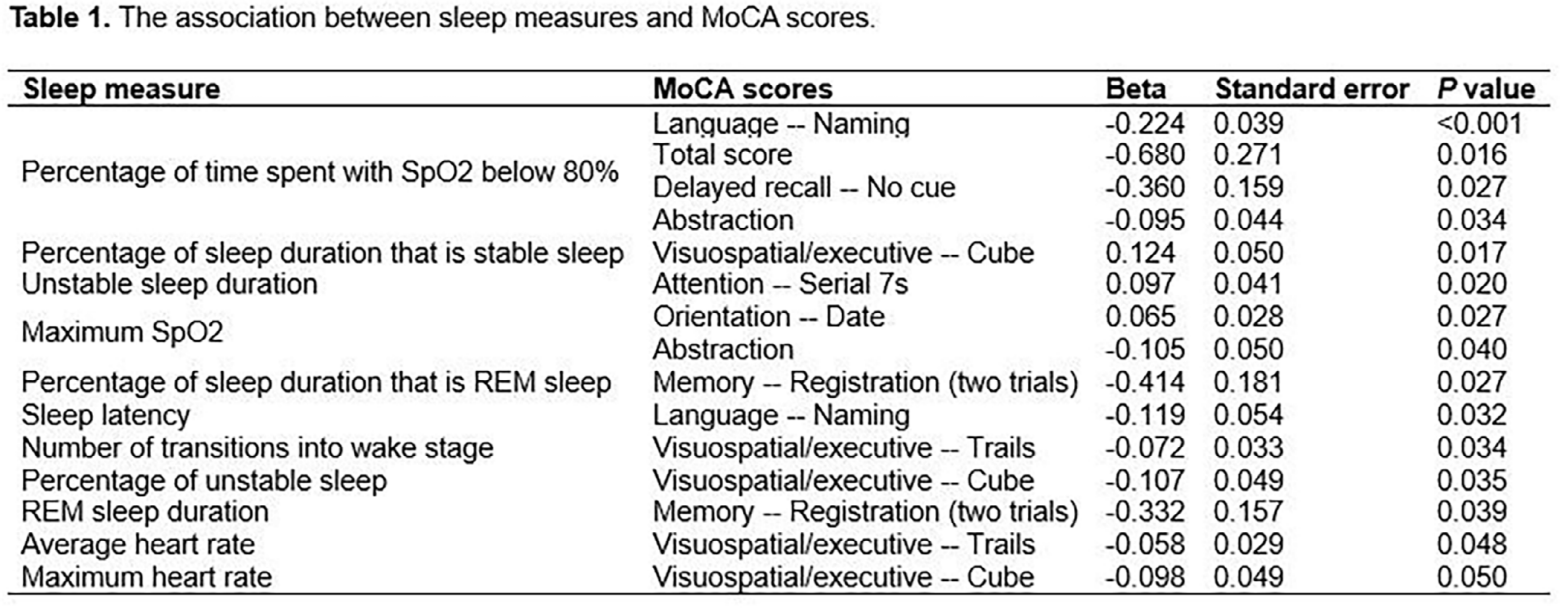# Association Between Sleep Measures and Cognition Performance: Insights from a Digital Sleep Assessment Study

**DOI:** 10.1002/alz.088924

**Published:** 2025-01-09

**Authors:** Huitong Ding, Chenglin Lyu, Edward Searls, Spencer Low, Zachary T Popp, Zexu Li, Salman Rahman, Akwaugo Igwe, Kristi Ho, Phillip H Hwang, Ileana De Anda‐Duran, Robert J Thomas, Vijaya B. Kolachalama, Rhoda Au, Honghuang Lin

**Affiliations:** ^1^ Boston University Chobanian & Avedisian School of Medicine, Boston, MA USA; ^2^ Boston University School of Public Heatlh, Boston, MA USA; ^3^ Tulane School of Public Health and Tropical Medicine, New Orleans, LA USA; ^4^ Beth Israel Deaconess Medical Center, Boston, MA USA; ^5^ University of Massachusetts Medical School, Worcester, MA USA

## Abstract

**Background:**

Sleep disorders as a contributing factor to cognitive impairment have spurred growing interest. The advent of digital technology facilitates the collection of comprehensive sleep measures in a home setting. The objective of this study is to examine the association between digital sleep measures and the Montreal Cognitive Assessment (MoCA).

**Method:**

This study included participants from the Boston University Alzheimer’s Disease Research Center (BU ADRC) Clinical Core, a longitudinal study of aging which includes the Uniform Dataset (UDS) and other Alzheimer’s Disease related clinical features. Participants were asked to wear a SleepImage Ring when they went to bed at night at least three times in a two week span at quarterly intervals. A variety of sleep measures, such as duration of unstable non‐rapid eye movement (NREM) sleep and percentage of time spent with SpO2 below 80%, were collected from the device and analyzed. Linear regression models were used to assess the associations between these sleep measures and the MoCA total score as well as individual MoCA scores. All models were adjusted for sex, age, and education to account for potential confounding factors.

**Result:**

Our study included 75 participants from the BU ADRC (mean age: 74.9± 7.9 years; 64.0% women). On average, the SleepImage Ring was worn for 17 nights over the duration of the study (interquartile range: 6‐23 nights). As shown in Table 1, 11 sleep measures were associated with at least one MoCA item with nominal significance (P<0.05). Interestingly, the percentage of time spent with SpO2 below 80% was negatively associated with the MoCA total score (P=0.016) as well as three individual MoCA scores, including the Language – Naming (P<0.001), Delayed recall – No cue (P=0.027) and Abstraction (P=0.034).

**Conclusion:**

Our analysis revealed multiple suggestive associations between digital sleep measures and MoCA test scores, highlighting the potential of sleep as a modifiable lifestyle factor to assess and improve cognitive health. Further studies with larger and independent samples are required to further validate these findings.